# Locally optimal extracellular stimulation for chaotic desynchronization of neural populations

**DOI:** 10.1007/s10827-014-0499-3

**Published:** 2014-06-05

**Authors:** Dan Wilson, Jeff Moehlis

**Affiliations:** Department of Mechanical Engineering, University of California, Santa Barbara, CA 93106 USA

**Keywords:** Parkinson’s disease, Lyapunov exponent, Optimal control theory

## Abstract

We use optimal control theory to design a methodology to find locally optimal stimuli for desynchronization of a model of neurons with extracellular stimulation. This methodology yields stimuli which lead to positive Lyapunov exponents, and hence desynchronizes a neural population. We analyze this methodology in the presence of interneuron coupling to make predictions about the strength of stimulation required to overcome synchronizing effects of coupling. This methodology suggests a powerful alternative to pulsatile stimuli for deep brain stimulation as it uses less energy than pulsatile stimuli, and could eliminate the time consuming tuning process.

## Introduction

Pathological synchronization among bursting neurons in the basal ganglia-cortical loop within the brain has been hypothesized to play a contributing role in the tremors seen in patients with Parkinson’s disease (Nini et al. 1995; Brownand and Marsden 1998; Levy et al. 2000; Chen et al. 2007; Pogosyan et al. 2010; Wichmann et al. 2011). Deep Brain Stimulation (DBS) is a well-established technique for alleviating these tremors. While the functional mechanism of DBS is not well understood, theoretical and experimental work suggest that DBS might desynchronize these pathologically synchronized neurons through the injection of high-frequency, pulsatile input into an appropriate region of the brain (Tass 2007; Hammond et al. 2007; Wilson et al. 2011). Viewing the problem of treating Parkinson’s disease through this lens has led to novel treatments which have been successful in primates (Tass 2003; Tass et al. 2012). Theoretical work, e.g., Rosenblum and Pikovsky (2004) and Popovych et al. (2005), show that desynchronization can be achieved with delayed feedback control to counter the effects of mean field coupling in a heterogeneous ensemble of oscillators. In Danzl et al. (2009), a minimum time desynchronizing control based on phase resetting for a coupled neural network was established using a Hamilton-Jacobi-Bellman approach, which was later extended by Nabi et al. (2013) to desynchronizeneurons using an energy-optimal criterion.

Typically, DBS is implemented with a high-frequency, pulsatile stimulus, and because each patient with Parkinson’s disease is different, a time intensive tuning process is used to select appropriate stimulus parameters to best mitigate symptoms. Despite attempts to better understand and improve the *in vivo* tuning process (Kuncel and Grill 2004; Volkmann et al. 2006), it remains somewhat of an art form, and the resulting stimulus is not guaranteed to be optimal for alleviating Parkinson’s symptoms. Furthermore, pulsatile stimuli are just a small subset of all stimuli that can be administered by a signal generator. This has motivated researchers to search for alternative stimuli that consume less energy in order to prolong battery life and to mitigate potential side effects of DBS including speech problems, difficulty swallowing, and motor contraction (Benabid et al. 2009). Furthermore, alternative stimuli may reduce the need to progressively increase the DBS voltage over time.

In Wilson and Moehlis (2014) we proposed a procedure for finding an energy-optimal stimulus which gives a positive Lyapunov exponent, and hence desynchronization, for a neural population. Unlike other proposed methods such as Danzl et al. (2009), Nabi et al. (2013), the procedure does not need the full model of the dynamics, and unlike Danzl et al. (2010), only requires a single input. Furthermore, this procedure is highly adaptable, and has the potential to be applied to be applied to other models of neural activity, such as those with bursting limit cycles oscillators (see Sherwood and Guckenheimer (2010)) which are now thought to play a crucial role in Parkinson’s disease (Bevan et al. 2006; Hahn et al. 2008; Gale et al. 2009; Ammari et al. 2011; Tai et al. 2011).

However, a major drawback of our method fromWilson and Moehlis (2014) is that it assumes charge-injection through each individual neural membrane using, for example, patch clamp techniques which are not feasible for neurons *in vivo*. Here we adapt the methodology to work for extracellular neural stimulation (as is the case for clinical DBS). We further adapt the model to limit Faradaic reactions that can be responsible for aggregate damage to the DBS probe and surrounding neural tissue for chronic DBS patients (Merill et al. 2005). The goal of this study is to move this methodology closer towards experimentation by providing numerical evidence that it can be implementedwithin a DBS framework.

In Section 2, we describe the model for extracellular stimulation. Section 3 defines the Lyapunov exponent for both a coupled and uncoupled system, and formulates a control problem to optimize the trade-off between relevant factors in the DBS stimulation such as total energy use, rate of desynchronization, and rate of Faradaic reactions. Section 4 gives results for this methodology applied to a model of thalamic activity. Section 5 gives concluding remarks, and Appendices A and B contain supporting simulations.

## Model of extracellular stimulation

We approximate the bursting regime in a fast-slow model of neural bursting (Ermentrout and Terman 2010) with a periodically spiking model of thalamic neurons (Rubin and Terman 2004):

1 $$ \dot{V_{i}} = \left[ I_{m,i} + I_{ext}(t) + \frac{1}{N} \sum^{N}_{i =1}\sigma_{ij}(V_{j}-V_{i}) + \eta_{i}(t) \right ] /C , $$

where

$$\begin{array}{@{}rcl@{}} I_{m,i} &=& -I_{L}(V_{i})-I_{Na}(V_{i},h_{i})-I_{K}(V_{i},h_{i})-I_{T}(V_{i},r_{i}) +I_{SM}, \\ \dot{h_{i}} &=& (h_{\infty}(V_{i})-h_{i})/\tau_{h}(V_{i}), \\ \dot{r_{i}} &=& (r_{\infty}(V_{i})-h_{i})/\tau_{r}(V_{i}), \quad i = 1,\dots, N. \end{array} $$

This is a conductance based model which reproduces the firing properties of a single population of thalamic neurons and has been used in previous modeling studies, for example, (Feng et al. 2007; Dorval et al. 2010; Schiff 2010). We emphasize that the following methodology is not limited to Eq. (1) and can be applied to any model for which the phase response curve can be found, as we have shown previously in Wilson and Moehlis (2014). We have augmented the voltage equation by additively including electrotonic coupling (Johnston and Wu 1995), DBS input, and Gaussian white noise. Here, *N* is the total number of neurons, *V*_*i*_,*h*_*i*_, and *r*_*i*_ are membrane voltage and gating variables for neuron *i*, *I*_*m*,*i*_ is the total membrane current for neuron *i*, *I*_*ext*_ is the external current from the electric field generated by the DBS probe, *σ*_*ij*_ characterizes the coupling strength between electrotonically coupled neurons *i* and *j*, with *σ*_*ij*_=*σ*_*ji*_ and *σ*_*ii*_=0 for all *i*, and $\eta _{i}(t)=\sqrt {2D}\mathcal {N}(0,1)$ is the i.i.d. noise associated with each neuron, assumed to be zero-mean Gaussian white noise with variance 2*D*. In this equation *I*_*SM*_ represents the baseline current which we take to be 5*μ*A/cm^2^ causing the neuron to fire with a period *T*=8.395 ms and *C*=1*μ* *F*/*c* *m* ^2^ is the constant neural membrane capacitance. For a full explanation of the functions *I*_*L*_,*I*_*Na*_,*I*_*K*_,*I*_*t*_,*h*_*∞*_,*τ*_*h*_,*r*_*∞*_ and *τ*_*r*_, we refer the reader to Rubin and Terman (2004).

**Fig. 1:**
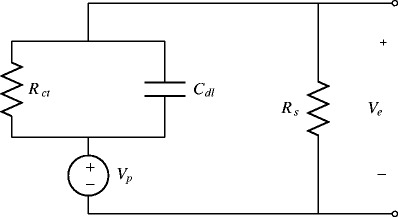
Equivalent circuit for extracellular stimulation

In order to characterize the external current, we must accurately describe the mechanisms of current flow through the extracellular fluid within the brain. The equivalent circuit for extracellular stimulation is shown in Fig. 1(Robinson 1968; Merill et al. 2005; Joye et al. 2009; Franks et al. 2005). The voltage source, *V*_*p*_, represents the voltage-controlled DBS probe. The mechanisms of charge transfer between the DBS electrode and the extracellular fluid fall into two categories: non-Faradaic and Faradaic. Non-Faradaic charge transfer can be modeled as charge flowing through an electrical capacitor, referred to as the double-layer capacitor, *C*_*dl*_ (Merill et al. 2005). This mechanism of charge transfer involves the transport of charged ions within the electrolyte, and is not typically associated with any harmful side effects. Often, the double-layer capacitance is represented as an equivalent impedance, *Z*=1/(*j* *ω* *C*_*dl*_)^*n*^, where *n* is a fitting parameter which usually takes values slightly smaller than 1. Here we represent *C*_*dl*_ as a true capacitance (i.e., *n*=1). The DBS electrode can also transfer charge through Faradaic oxidation and reduction reactions. These reactions are not always reversible, and are associated with corrosion of the DBS electrode and damage to the surrounding neural tissue. For relatively low levels of charge injection, the current from Faradaic reactions can be modeled as a resistance to charge transfer, *R*_*ct*_ (Merill et al. 2005). Current from the probe spreads through the extracellular medium creating an extracellular potential that obeys the Laplace equation,

2 $$ \nabla \cdot \sigma_{c} \nabla V = 0,  $$

where *V*(**z**) is the voltage at spatial position **z**, and *σ*_*c*_ is the conductivity of the extracellular medium, assumed to be uniform. The effect of this spreading can be treated as an equivalent resistance, *R*_*s*_ (Robinson 1968). We assume that the neurons within the extracellular fluid do not influence the circuit, allowing the equation for this circuit to follow from Kirchhoff’s current law,

3 $$ \dot{V_{e}} = \dot{V_{p}} + \frac{V_{p}-V_{e}}{C_{dl}R_{ct}} - \frac{V_{e}}{C_{dl} R_{s}},  $$

where *V*_*p*_−*V*_*e*_ is the voltage across the double layer capacitor, and *V*_*p*_ is the probe voltage.

The values of *C*_*dl*_, *R*_*ct*_, and *R*_*s*_ are dependent on many factors including probe size, geometry, material, and temperature, and will vary widely between *in vitro* settings, animal trials, and therapeutic DBS. For instance, the value of *C*_*dl*_ can be manipulated through the selection of the material, particularly using materials with surface oxide present can increase this value. The value of *R*_*s*_ can be directly calculated for some probe shapes (Robinson 1968), and generally shrinks as the size of the voltage probe grows. The parameter *R*_*ct*_ depends on the reactions between the probe and the extracellular medium. In this paper, we take *C*_*dl*_=5×10^−4^ *F*, *R*_*s*_=29Ω, and *R*_*ct*_=2×10^5^Ω to be physiologically reasonable values which lie between the properties of platinum and platinum black based on experimental recordings from Franks et al. (2005). We note that qualitative results are not dependent on the exact choices of these parameters, but we have found numerically that a larger value of *C*_*dl*_ relative to *R*_*ct*_ is a design parameter which allows for better control of *V*_*e*_, since current does not spread through the extracellular medium as quickly.

**Fig. 2:**
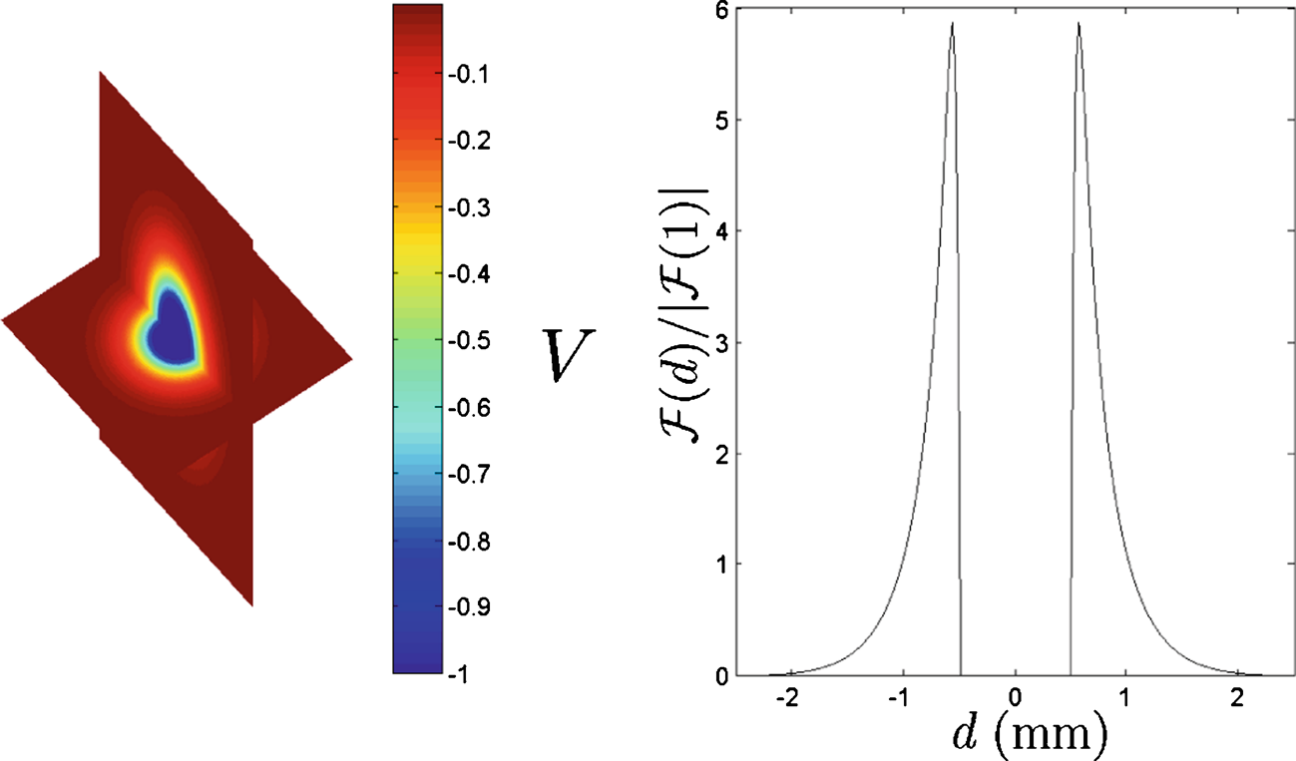
The *left panel* shows the solution of Eq. (2) for a spherical probe with 1 mm diameter with *V*_*e*_=−1V. The *right panel* shows $\frac {\partial ^{2} V}{\partial x^{2}}$ for *x* perpendicular to an imaginary line extending from the center of the probe to the neuron

The effective current flow along each neuronal process (i.e. axon or dendrite) is proportional to the second spatial derivative of the voltage along each process, i.e., $\frac {\partial ^{2} V}{\partial z^{2}}$, where *z* points in the direction of the neural process (Rattay 1986; McIntyre et al. 2004). Rather than examining each neural process individually with a compartmental model, we note that DBS has the strongest influence on the axon of each neuron (Wu et al. 2001; Nowak and Bullier 1998; McIntyre et al. 2004), so that the effective current flow into the neuron is proportional to $\frac {\partial ^{2} V}{\partial x^{2}}$, where *x* points in the direction of the neuron’s axon. Furthermore, we assume that *x* is the same for all neurons, which represents a worst case scenario because a distribution in *x* among neurons creates a distribution of effective inputs and hastens desynchronization, as illustrated in Appendices A and B. Without loss of generality, we take *x* perpendicular to a line extending from the center of the probe to each neuron. The potential generated in the extracellular medium is calculated according to Eq. (2) and is shown in Fig. 2 for a spherical probe with 1 mm diameter and *V*_*e*_, the voltage at the tip of the probe, equal to -1V.

For simplicity of notation, let

4 $$ \mathcal{F}(d) = \frac{\partial^{2} V}{\partial x^{2}}(d) \quad \text{when} \quad V_{e} = -1\mathrm{V}, $$

where *d* is the distance from the neuron to the center of the probe. We note that $\mathcal {F}(d)$ is an approximation to the effective stimulus the neurons receive when the voltage at the tip of the probe is -1V. Note that the solution of Eq. (2) can be scaled by a positive constant, meaning that the magnitude of the effective strength plot in Fig. 2 scales with *V*_*e*_. Effective extracellular inputs can be characterized as in Rattay (1986)

5 $$ I_{ext}(t) = \frac{\partial^{2} V}{\partial {x}^{2}} \frac{1}{R_{i}} = \frac{ -\mathcal{F}(d) V_{e}} {R_{i}}, \label {iext} $$

where *R*_*i*_ is the effective intra-axonal resistance.

## Optimal control of Lyapunov exponents

Ultimately, we wish to find a control, *I*_*ext*_(*t*) which has the effect of desynchronizing the neural network given by Eq. (1). In order to simplify the model of a periodically spiking neuron from Eq. (1), we employ the phase reduction following e.g., Brown et al. (2004),

6 $$ \frac{d \theta}{dt} = \omega + Z(\theta)u_{e}(t),  $$

where *𝜃* is the phase of the neuron and is 2*π* periodic on [0,2*π*). By convention *𝜃*=0 corresponds to the time at which the neuron spikes. Here, *ω* gives the neuron’s baseline dynamics, determined from its natural period *T* as *ω*=2*π*/*T*, *Z*(*𝜃*) is the neuron’s phase response curve (PRC), and

7 $$ u_{e}(t) \equiv \frac{I_{ext}(t)}{C} = \frac{ -\mathcal{F}(d) V_{e}(t)} {R_{i}C} $$

represents the control input from the electric field. Previously, we derived an expression for the finite time Lyapunov exponent, Λ(*τ*) for a particular signal to be Wilson and Moehlis (2014)

8 $$ \Lambda(\tau) = \frac{ \int_{a}^{a+\tau} Z'(\theta (s))u(s)ds}{\tau} , $$

where ^′^ = *d*/*d* *𝜃*. The finite time Lyapunov exponent describes the exponential divergence of two identical neurons with two similar initial conditions. Larger values of Λ>0 correspond to a faster desynchronizing influence of the applied signal. Recall from Section 2 that we only have control over the probe voltage, *V*_*p*_, which influences the circuit according to Eq. (3). In order to conveniently compare the neuron’s response to the electric field to the control input at the probe, we define

9 $$ u_{p}(t) \equiv \frac{-\mathcal{F}(d) V_{p}(t)} {R_{i}C} $$

as the voltage-controlled probe input. Note that because *u*_*p*_ and *u*_*e*_ are merely constant scalings of *V*_*p*_ and *V*_*e*_, Eq. (3) still applies. In the analysis that follows, we assume that all neurons are located at *d*=1mm from the probe so that $\mathcal {F}(d) / \mathcal {F}(1)= 1$. Clearly, in a more realistic setting, there will be a heterogeneous distance and position distribution of neurons from the probe, but ultimately, as we provide evidence for in Appendix A, a homogeneous distribution of neurons represents a worst case scenario in terms of desynchronization.

Suppose we want to find the stimulus that minimizes the cost function

10 $$\begin{array}{@{}rcl@{}} \mathcal{G}[V_{p}(t)] &&= \int_{0}^{t_{1}} [(u_{e}(t))^{2} + \alpha(\dot{ u}_{p}(t))^{2} - \beta Z'(\theta)u_{e}(t)\notag\\ &&{\kern10pt}+ \gamma (u_{p}(t) - u_{e}(t) )^{2} ]dt. \end{array} $$

Here, $ \int _{0}^{t_{1}} [u_{e}(t)^{2}]dt$ is proportional to the square of the current created by the voltage source, and hence the power consumed by the stimulus, $\int _{0}^{t_{1}} [\dot {u}_{p}(t)^{2}]dt $ is a quantity that limits the rate at which the probe voltage can vary and is necessary so that the optimal control problem is not singular (Kirk 1998), $\int _{0}^{t_{1}} [(u_{p} - u_{e} )^{2}]dt $ represents the Faradaic charge transfer, assuming that all Faradaic reactions are irreversible, and *α*, *β*, and *γ* are positive constants used to determine the relative importance of each term. We note that a true approximation of Faradaic current would be $\int _{0}^{t_{1}} | u_{p} - u_{e} |dt $, but for computational reasons, we use $ \int _{0}^{t_{1}} [(u_{p} - u_{e} )^{2}]dt $ to accomplish the same goal of keeping Faradaic current low. We apply calculus of variations (Kirk 1998) to minimize $\mathcal {C}[\boldsymbol {\Phi }(t),\dot {\boldsymbol {\Phi }}(t), u_{p}(t)] = \int _{0}^{t_{1}} \mathcal {M}[u_{p}(t)]dt$ where

11 $$\begin{array}{@{}rcl@{}} \mathcal{M}[u_{p}(t)] &&= (u_{e}(t))^{2} + \alpha (\dot{ u}_{p}(t))^{2} - \beta Z'(\theta)u_{e}(t) \\ &&{\kern8pt} + \gamma (u_{p}(t) - u_{e}(t))^{2}\\ &&{\kern8pt}+ [\lambda_{1} {} \quad \lambda_{2}] \left[\begin{array}{cc} \dot{u}_{e} - \dot{u}_{p} - \frac{u_{p}-u_{e}}{C_{dl}R_{ct}} + \frac{u_{e}}{C_{dl} R_{s}} \\ \dot{\theta} - \omega - Z(\theta)u_{e} \end{array} \right], \end{array} $$

and ***Φ***(*t*)=[*𝜃*,*u*_*e*_(*t*),*λ*_1_,*λ*_2_]^*T*^. The Lagrange multipliers *λ*_1_ and *λ*_2_ force the dynamics to satisfy Eqs. (3) and (6). The associated Euler-Lagrange equations are

12 $$ \frac{\partial \mathcal{M}}{\partial u_{p}} = \frac{d}{dt}\left(\frac{\partial \mathcal{M}}{\partial \dot{u}_{p}} \right), \quad \frac{\partial \mathcal{M}}{\partial \boldsymbol{\Phi}} = \frac{d}{dt}\left(\frac{\partial \mathcal{M}}{\partial \boldsymbol{\dot{\Phi}}} \right), $$

which yield the set of ordinary differential equations, written in standard form:

$$ \left[\begin{array}{cccccc} 1 & 0 & 0 & 0 & 0 & 0 \\ 0 & 2 \alpha & 0 & 0 & -1 & 0 \\ 0 & 0 & 0 & 0 & 0 & 1 \\ 0 & 0 & 0 & 0 & 1 & 0 \\ 1 & 0 & 0 & -1 & 0 & 0 \\ 0 & 0 & 1 & 0 & 0 & 0 \end{array}\right] \left[\begin{array}{c} \dot{X_{1}} \\ \dot{X_{2}} \\ \dot{\theta} \\ \dot{u_{e}} \\ \dot{\lambda_{1}} \\ \dot{\lambda_{2}} \end{array}\right] =\left[\begin{array}{c} X_{2} \\ 2 \gamma(X_{1}-u_{e}) - \frac{\lambda_{1}}{C_{dl}R_{ct}} \\ -(\beta Z^{\prime\prime}(\theta) + \lambda_{2} Z'(\theta))u_{e} \\ 2u_{e} - \beta Z'(\theta) - 2\gamma(X_{1}-u_{e}) - \lambda_{2} Z(\theta) + \lambda_{1}(\frac{1}{C_{dl}R_{ct}} + \frac{1}{C_{dl}R_{s}}) \\ \frac{u_{e}}{C_{dl}R{s}} -\frac{X_{1}-u_{e}}{C_{dl}R_{ct}} \\ \omega + Z(\theta)u_{e} \end{array}\right], $$

black where *X*_1_=*u*_*p*_, and $X_{2} = \dot {u_{p}}$. We solve Eq. (13) subject to starting boundary conditions *X*_1_(0)=*X*_2_(0)=*𝜃*(0)=*u*_*e*_(0)=0 and end-point boundary conditions *X*_1_(*t*_1_)=*u*_*e*_(*t*_1_)=0. This can be done numerically by finding an initial condition for the Lagrange multipliers *λ*_1_ and *λ*_2_ that satisfies the boundary conditions, which is solved using a double bisection algorithm as described in Danzl et al. (2010). We note that this methodology provides a locally optimal solution for the control input *u*_*e*_(*t*) and does not preclude the existence of a different stimulus that is globally optimal. However, for all results presented in this paper, we search for plausible solutions with initial Lagrange multipliers until they yield control inputs with large control inputs that invalidate the phase reduction Eq. (6), i.e., the control inputs become so large that they take the system far from the limit cycle.

If we have knowledge of how the neurons are coupled to each other, and if the influence of the intercellular coupling is similar to that of the extracellular stimulation, we can include the influence of coupling in the optimization process. Consider two deterministic neurons from Eq. (1) which are similar in phase so that *𝜃*_1_≈*𝜃*_2_≡*𝜃*. The phase reduction for these two neurons within the larger population of *N* neurons is given by

13 $$\begin{array}{@{}rcl@{}} \frac{d \theta_{j}}{dt} &=& \omega + Z(\theta_{j}(t))u(t)+ \frac{1}{N} Z(\theta_{j}(t)) \sum_{i=1}^{N} \sigma_{ij}(f(\theta_{i}(t))\\ &&-f(\theta_{j}(t))), \quad j = 1,2, \end{array} $$

where *f*(*𝜃*) gives the transmembrane voltage as a function of *𝜃* assuming the trajectory is on the periodic orbit. We take the coupling to be all-to-all with *σ*_*ij*_=*σ* for all *i*≠*j*. Letting *ϕ*=|*𝜃*_2_−*𝜃*_1_|, we obtain from the Taylor expansion

14 $$\begin{array}{@{}rcl@{}} \frac{d \phi}{dt} &=& \left[ Z'(\theta) \left(u + \sigma \sum_{i = 1}^{N} (f(\theta_{i})-f(\theta)) \right)\right.\\ &&- \left.\frac{N-1}{N} \sigma f'(\theta) Z(\theta) \right] \phi+ \mathcal{O}(\phi^{2}), \end{array} $$

where ^′^ = $\frac {d}{d\theta }$. Note that we have dropped explicit time dependence for simplicity of notation. We assume solutions of the form *ϕ*∼*e* ^Λ*t*^ and further simplify Eq. (14) by assuming *N* is large enough so that $\frac {N-1}{N} \approx 1$ to yield the finite time Lyapunov exponent

15 $$ \Lambda_{c}(\tau) =\frac{ \log(\phi(\tau))}{\tau} = \frac{ \int_{a}^{a + \tau} \left[Z'(\theta) \left(u + \sigma \sum_{i = 1}^{N} (f(\theta_{i})-f(\theta)) \right) - \sigma f'(\theta) Z(\theta) \right] dt}{\tau}. $$

The critical difference between Eq. (8) and (15) is that Λ_*c*_ provides a precise measure of the desynchronizing effect of the control signal. A signal yielding Λ_*c*_(*t*)>0 will guarantee desynchronization for the coupled system, whereas Λ(*t*) must be *large enough* to counteract coupling in order to desynchronize the system; this immediately leads to the question, “how large is large enough?” that can only be answered *ad hoc* as in Wilson and Moehlis (2014).

For the model Eq. (1), we find that *f* ^′^(*𝜃*)*Z*(*𝜃*)>0 for most values of *𝜃*, meaning that for positive values of *σ*, the electrotonic coupling serves to reduce the overall Lyapunov exponent. In the absence of external control, Λ_*c*_ will be negative, serving to exponentially synchronize the system.

Equation (15) is difficult to use within the framework of calculus of variations, because it assumes explicit knowledge of the phase distribution. However, if we focus on desynchronizing neurons that are close to the maximum of the distribution, and assume that the distribution is symmetric around the maximum, we find numerically that ${\sum _{i}^{N}} (f(\theta _{i})-f(\theta )) \approx 0$, except when *𝜃*≈2*π*. Fortunately, for many neural models, *Z* ^′^(2*π*) is small, and thus, we can accurately approximate the Lyapunov exponent as

16 $$ \Lambda_{c}(\tau) \approx \frac{\int_{a}^{a + \tau} \left [ Z'(\theta) u - \sigma f'(\theta) Z(\theta) \right ]dt}{\tau}. $$

Using Eq. (16), it is simple to recast the optimal control problem to account for interneuronal coupling. We applycalculus of variations to minimize $\mathcal {G}[\boldsymbol {\Phi }(t),\dot {\boldsymbol {\Phi }}(t), u_{p}(t)]= \int _{0}^{t_{1}} \left [ \mathcal {M}[u_{p}(t)] + \sigma f' \right .$ (*𝜃*)*Z*(*𝜃*)]*d* *t* where $\mathcal {M}[u_{p}(t)]$ was defined as part of our original control problem in Eq. (11). The new set of Euler-Lagrange equations, written in standard form are

17 $$ \left[\begin{array}{cccccc} 1 & 0 & 0 & 0 & 0 & 0 \\ 0 & 2 \alpha & 0 & 0 & -1 & 0 \\ 0 & 0 & 0 & 0 & 0 & 1 \\ 0 & 0 & 0 & 0 & 1 & 0 \\ 1 & 0 & 0 & -1 & 0 & 0 \\ 0 & 0 & 1 & 0 & 0 & 0 \end{array}\right] \left[\begin{array}{c} \dot{X_{1}} \\ \dot{X_{2}} \\ \dot{\theta} \\ \dot{u_{e}} \\ \dot{\lambda_{1}} \\ \dot{\lambda_{2}} \end{array}\right] = \left[\begin{array}{c} X_{2} \\ 2 \gamma(X_{1}-u_{e}) - \frac{\lambda_{1}}{C_{dl}R_{ct}} \\ -(\beta Z^{\prime\prime}(\theta) + \lambda_{2} Z'(\theta))u_{e} + \beta\sigma(f^{\prime\prime}(\theta)Z(\theta) + f'(\theta)Z'(\theta)) \\ 2u_{e} - \beta Z'(\theta) - 2\gamma(X_{1}-u_{e}) - \lambda_{2} Z(\theta) + \lambda_{1}(\frac{1}{C_{dl}R_{ct}} + \frac{1}{C_{dl}R_{s}}) \\ \frac{u_{e}}{C_{dl}R{s}} -\frac{X_{1}-u_{e}}{C_{dl}R_{ct}} \\ \omega + Z(\theta)u_{e} \end{array}\right]. $$

Note the subtle difference between Eq. (13) and (17), which differ by extra terms in the third row of the right hand side.

## Results and discussion

### Neglecting the influence of interneuron coupling in calculating the optimal stimulus

All calculations from Section 4 were performed with basic parameters listed in Table 1. It may be difficult to accurately characterize the interneuron coupling for control purposes. Here, we show that even if the coupling function is unknown, we can still solve for stimuli that can desynchronize a pathologically synchronized neural network. For a single neuron described by Eq. (1), the baseline current causes it to spike with a period *T*=8.395ms. To obtain the optimal control, we take *t*_1_=8.02ms (corresponding to *𝜃*=6.0 on the limit cycle), *β*=50, *α*=0.2 and a range of different values of *γ*. We take *t*_1_ slightly smaller than the full period of a single neuron. If we were to take *t*_1_ to be the full period of a neuron, it is more likely for neurons to spike before the optimal stimulus is finished. Note that *t*_1_ can be chosen differently provided it is sufficiently smaller than *T*.

**Table 1:** Basic set of parameters used for simulation in Section 4

Parameter	Symbol	Nominal Value
Neural Membrane Capacitance	*C*	1 *μ*F/ cm^2^
Baseline current	*I*_*SM*_	5 *μ*A/ cm^2^
Electrotonic coupling strength	*σ*	0.07
Gaussian white noise variance	2*D*	0.7
Spreading resistance	*R*_*s*_	29Ω
Charge transfer resistance	*R*_*ct*_	2×10^5^Ω
Double layer capacitance	*C*_*dl*_	5×10^−4^ F
Neural distance from voltage probe	*d*	1 mm
Probe voltage derivative weighting parameter	*α*	0.2
Lyapunov exponent weighting parameter	*β*	50
Faradaic current weighting parameter	*γ*	8
Optimal stimulus duration	*t*_1_	8.02 ms

**Fig. 3:**
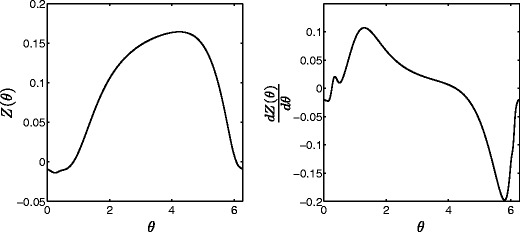
*Left* and *right* panels show the PRC and its first derivative for a single neuron from Eq. (1)

**Fig. 4:**
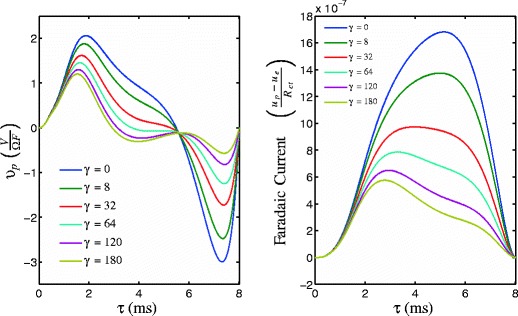
Optimal stimuli and Faradaic current for different values of *γ*. As *γ* increases, the current from the optimal stimulus becomes concentrated where the system is most susceptible to desynchronization, i.e. at the extrema of *Z* ^′^(*𝜃*)

The left and right panels of Fig. 3 show the PRC and its first derivative for a single neuron from Eq. (1), obtained numerically using the software X-Windows Phase Plane (XPP) (Ermentrout 2002). Figure 4 gives the obtained locally optimal stimuli obtained from solving Eq. (13) for varying values of *γ*. Lyapunov exponents for each signal as calculated from Eq. (8) are 0.097, 0.077, 0.054, 0.040, 0.028, and 0.021 for *γ*= 0, 8, 32, 64, 120, and 180, respectively. Recall that a larger value of *γ* corresponds to a smaller toleration for Faradaic current. We note that during each stimulation, *𝜃*(*t*)≈*ω* *t*, and it can be seen by comparing Fig. 4 to Fig. 3 that as *γ* increases, the current from the optimal stimulus becomes concentrated around the extrema of *Z* ^′^(*𝜃*), where the system is most susceptible to desynchronization. This result agrees with intuition that can be gained from the impedance properties of the circuit from Fig. 1. We find that

18 $$ \frac{(V_{p}-V_{e})(s)}{V_{s}(s)} = \frac{R_{ct}}{R_{ct}+R_{s}(R_{ct}C_{dl}s+1)}. $$

A quick pulse of current at *V*_*p*_ will be transmitted through the filter to *V*_*e*_ without much loss, while a slower pulse will lose much of its amplitude through the filter. Since Faradaic current is proportional to *V*_*p*_−*V*_*e*_, a quicker pulse will create less Faradaic current.

**Fig. 5:**
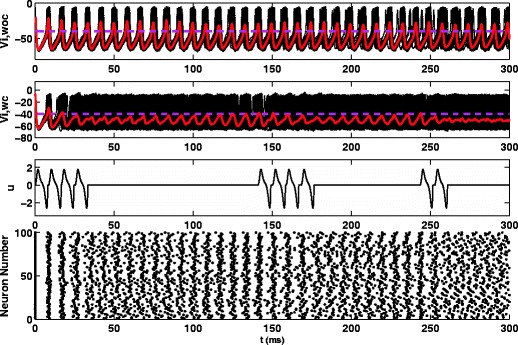
Results for a population of *N*=100 noisy, coupled neurons. The *first panel* shows results in the absence of control. The *second* and *third panels* show results for the same network with the event-based control applied. Traces give the mean voltages for the system and the horizontal line shows the control activation threshold. Substantial desynchronization can be seen from the raster plot

We now apply the optimal control found for *γ*=8 to a network of *N*=100 coupled, noisy neurons with identical coupling *σ*_*ij*_=0.07 and i.i.d. noise with *D*=0.7 in order to test the desynchronizing effects on the full model. We define the mean voltage as our system observable, $\overline {V}(t) = \frac {1}{N} \sum ^{N}_{i=1} V_{i}(t)$. The controller has two states: *active* and *inactive*. When the controller is active, a new stimulus is triggered when $\overline {V}>-45\rm {mV}$ and $\dot {\overline {V}} < 0$. Once $\overline {V}$ no longer spikes above -45mV, the controller switches to an *inactive* state. It changes back to an *active* state if $\overline {V}$ registers above -40mV. We call this event-based control because the controller is only triggered when the mean voltage crosses a certain threshold. We use the algorithm presented in Honeycutt (1992) to simulate the noisy system. Figure 5 shows the results of this simulation. The top panel shows that the network remains synchronized in the absence of control. The second panel shows the voltage traces of each neuron and the average voltage for a network with the optimal control applied according to the algorithm given above. The third panel shows the control input over time. We find that the optimal control is effective at keeping the average voltage of the network below the target voltage, shown as a horizontal line, and must only apply a few stimuli approximately once every 100ms. The desynchronizing effect of the stimulus can clearly be seen from the raster plot.

**Fig. 6:**
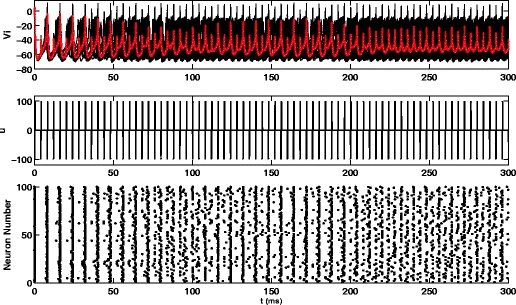
The *top* and *middle panels* show results for a population of *N*=100 noisy, coupled neurons with a pulsatile control. Traces give the mean voltages for the system. The raster plot shows that the pulsatile stimulus desynchronizes by splitting the network into two groups of neurons firing out of phase

For comparison, we show the results for a network with a continuously applied, pulsatile stimulus as is currently used in DBS to treat Parkinson’s disease. The stimulus is charge-balanced and biphasic, with a pulse width of 0.2ms for each phase, a period of 4 ms giving a frequency of approximately twice the firing rate of the neurons, and an amplitude of *u*_*p*_=98*V*/Ω*F*. These parameters were found by systematically simulating Eq. (1) with pulsatile stimuli of varying amplitudes, frequencies, and pulse widths and evaluating the desynchronization in the network. We note that this strategy of finding an effective desynchronizing stimulus is not unlike the time intensive process of tuning DBS parameters to an individual patient with Parkinson’s disease and that this is the best combination of parameters found using this strategy based on overall efficacy of the stimulus and stimulus magnitude. The top panel of Fig. 6 shows the individual voltage traces of each neuron as well as the average voltage of the network, and the second panel shows the control input over time. The pulsatile stimulus desynchronizes the network by eventually separating the neurons into two groups, firing out of phase with each other.

**Fig. 7:**
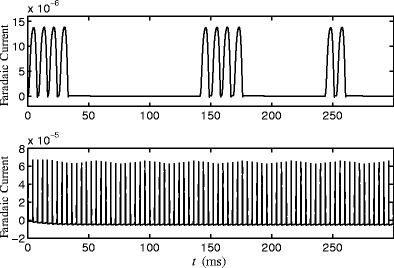
The *top* and *bottom panels* show Faradaic current (measured as (*u*_*p*_−*u*_*e*_)/*R*_*ct*_) from the simulations shown in Figs. 5 and 6, respectively

For long-term DBS stimulation for treatment of Parkinson’s disease, the total energy used by the DBS probe and rate of Faradaic reactions are important considerations for longevity of the implanted battery, and aggregate damage to the DBS probe and surrounding neural tissue. Throughout each 300ms simulation, the total energy injected into the brain is calculated as $\int {u_{e}^{2}} dt$ and is found to be 124 and 304000 units for the optimal stimulus and the pulsatile stimulus respectively. While both stimuli are able to cause network desynchronization, the optimal stimulus does so with 3 orders of magnitude less energy injected into the brain, representing a tremendous savings in energy. We also consider Faradaic reactions for each simulation, shown in Fig. 7. The total Faradaic current for the optimal stimulus and pulsatile stimulus, calculated as $\int |u_{p}-u_{e}| / R_{ct} dt$, is 0.00067 and 0.00224 units, respectively. The total Faradaic charge transfer is similar for both stimuli, which is initially surprising. The optimal stimulus is applied over a longer time scale than a pulsed stimulus, which, according to the transfer function from Eq. (18), should result in large amounts of Faradaic current. However, for the pulsatile stimulus, the value *u*_*p*_−*u*_*e*_ begins to drift downward when no energy is being applied by the controller, creating a small, but persistent, Faradaic current. This drift is not as prevalent in the simulation for the optimal stimulus because we required that *u*_*e*_=*u*_*p*_ in the solution to the optimization Eq. (13). Also, the magnitude of the optimal stimulus is much smaller than the pulsatile stimulus, which further reduces Faradaic charge transfer. Overall, we find that the optimal stimulus desynchronizes the network by injecting much less energy into the brain and using a comparable amount of Faradaic charge as a pulsatile stimulus.

It is worth noting that in Section 2 we assumed that all neurons were equidistant and oriented in the same way in reference to the probe. In reality, there will be a distribution of distances from the probe of each neuron, which will manifest in different values of $\mathcal {F}(d)$ for each neuron’s external current from the DBS probe. Fortunately, as might be expected by from the work of Winfree, (Winfree 1967; 2001), heterogeneity in the distances from the probe, leading to heterogeneity in the effective inputs, only serves to hasten desynchronization of a neural network Eq. (1) (see Appendices), and the formulation from Section 2 represents a worst case scenario.

### Accounting for coupling in calculating the optimal stimulus

**Fig. 8:**
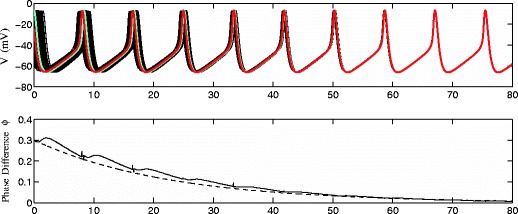
The *top panel* shows a network Eq. (1) in the absence of noise and external stimuli. The *black lines* show the voltage traces of each individual neuron in the network. We monitor the phase of two individual neurons, shown as red and blue traces in the *top panel*. The numerically obtained phase difference (*solid line*) and expected phase difference (*dashed line*) calculated from Λ_*c*_ Eq. (16) are in good agreement

We have found that if we do not take coupling into account, either because it is difficult to characterize mathematically or we simply do not know how the neurons are coupled, the optimization procedure can still give a stimulus that is quite effective at desynchronizing a pathologically synchronized population of neurons. But if we know that the interneuron coupling is electrotonic, we can give a more precise measurement of the Lyapunov exponent Eq. (15). First, in order to verify the validity of the approximation of the Lyapunov exponent with coupling given by Eq. (16), we simulate the system Eq. (1) in the absence of noise or external stimulation. Choosing the coupling strength to be *σ*=0.07 we expect Λ_*c*_(*T*)=0.0443, calculated from Eq. (16). Figure 8 shows the result of this simulation. As expected, we find in the top panel that the coupling synchronizes the identical neurons until they are nearly in phase. We also infer the phase of two different neurons (shown as red and blue traces in the top panel) at each time step by simulating each neuron separately in the absence of DBS input and noise to determine when it spikes next. The numerically determined phase difference is shown as a solid line in the bottom panel of Fig. 8, and the expected phase difference based on the calculated Lyapunov exponent from Eq. (16) is shown as a dashed line. We see good agreement between the expected and numerical phase differences.

**Fig. 9:**
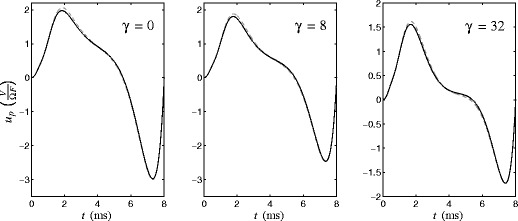
Comparing the solution to Eq. (13) and Eq. (17), shown as dashed and solid lines, respectively, for different values of *γ*. We find that the two solutions are nearly indistinguishable

The locally optimal stimuli from Section 4.1 minimized the cost function Eq. (11) where coupling was not taken into account. Here, we would like to know if we can find an even better stimulus when we know the coupling explicitly. We solve Eq. (17) with the same boundary conditions and parameters as before to investigate how the solution changes when we account for coupling. We find that including the influence of coupling in the optimization has relatively little impact on the obtained solution. For instance, Fig. 9 shows the solution to Eq. (17) for various values of *γ*. The corresponding optimization without considering coupling is shown as a gray, dashed line for reference. Including coupling in the optimization yields a slightly larger bias towards positive stimulus when *𝜃*≈4.5, corresponding to the peak of the PRC, shown in Fig. 3. This positive bias acts to speed up the dynamics when the synchronizing effects of coupling are strongest. However, the overall solutions to Eq. (13) and (17) are nearly indistinguishable. It is much more energy-efficient to desynchronize by exploiting the phase model dynamics than it is to mitigate the effect of interneural coupling for this network model of thalamic neurons.

**Fig. 10:**
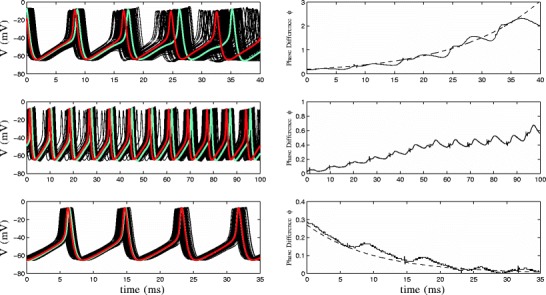
The *top*, *middle* and *bottom panels* show simulations of Eq. (1) without noise for stimuli with Λ_*c*_=0.0286,−0.0077, and −0.0253, respectively. *Left panels* show voltage traces for each neuron, and *right panels* show *ϕ*(*t*) for the highlighted neurons in the *left panels*, as well as exponential functions fit to the data. We find that Λ_*c*_ underestimates the overall rate of desynchronization for each stimulus. However, calculation of Λ_*c*_ allows for qualitative prediction of the overall desynchronizing capabilities of a stimulus. For Λ_*c*_ sufficiently larger than 0, we should see marked exponential desynchronization. For Λ_*c*_ sufficiently smaller than 0, coupling will dominate, and we will not have desynchronization. For Λ_*c*_≈0, we may or may not see desynchronization, but *ϕ*(*t*) may not be characterized by an exponential function

For the thalamus model Eq. (1), including coupling in the formulation of the optimal control problem does not significantly change the resulting signal. However, the ability to more precisely characterize the overall rate of desynchronization with Λ_*c*_ is much more useful. Table 2 lists the values of Λ(*T*) and Λ_*c*_(*T*) for some of the stimuli shown in Fig. 4. Based on the derivation of Eq. (16), for Λ_*c*_, we to expect be able to quantitatively predict the rate of desynchronization of a system of neurons for a given stimulus. However, in practice we find that in phase space, the neurons stray from the periodic orbit when the optimal control is applied, leading to small changes in the value of *f*(*𝜃*), the transmembrane voltage as a function of *𝜃* assuming the trajectory is on the periodic orbit, and influences the effective strength of the coupling. For Eq. (1), this has the effect of decreasing the effect of coupling when the phase differences are small. However, Fig. 10 shows that we can still make qualitative predictions about the overall desynchronizing ability of a stimulus. The top, middle, and bottom panels show simulations of Eq. (1) without noise for stimuli from Fig. 4 with *γ*=8, 64, and 180, respectively. Left panels show voltage traces for each neuron, and right panels show the phase difference *ϕ*(*t*) for the neurons highlighted in the left panels, as well as exponential functions fit to the data if applicable. For the stimulation in the top panel, Λ_*c*_=0.0286, and we find that neurons exhibit a strong overall exponential desynchronization, with a numerically calculated Lyapunov exponent of 0.069. The middle panel shows results using a stimulus with Λ_*c*_=−0.0077. For this simulation, the neurons do desynchronize, but the desynchronization is clearly not exponential. The bottom panel shows results for a stimulus with Λ_*c*_=−0.0253, which yields desynchronization with a numerically determined Lyapunov exponent of -0.010. In each case, Λ_*c*_ underestimates the desynchronizing capability of the stimulus, but can give a good qualitative prediction of whether a given stimulus can desynchronize the system. For Λ_*c*_ sufficiently larger than 0, we should see strong exponential desynchronization. For Λ_*c*_ sufficiently smaller than 0, coupling will dominate, and we will not have desynchronization. For Λ_*c*_≈0, we may or may not see desynchronization, and *ϕ*(*t*) may not be characterized by an exponential function. In Appendix B, we show similar desynchronizing effects of our stimuli when neurons are synaptically coupled.

**Table 2:** Stimulus properties from Fig. 9

*γ*	Λ(*T*)	Λ_*c*_(*T*)	Numerically determined Λ
8	0.0773	0.0286	0.0690
64	0.0399	−0.0077	Desynchronization Not Exponential
180	0.0214	−0.0253	−0.010

## Conclusion

In this paper, we present an adaptation of the methodology presented in Wilson and Moehlis (2014) to locally, optimally maximize the Lyapunov exponent Eq. (8) while taking into account factors such as overall energy used and Faradaic charge transfer for a model of extracellular stimulation. We have also investigated the effects of electrotonic coupling on a large system of neurons in an attempt to more precisely characterize the rate of desynchronization as determined by a Lyapunov exponent. We find from numerical simulation that this methodology provides a locally optimal stimulus that has the ability to desynchronize a population of neurons by injecting three orders of magnitude less energy in to the brain when compared to a pulsatile stimulus while generating similar levels of Faradaic charge transfer. Furthermore, the implementation of this methodology only requires knowledge of a system’s phase response curve, which is experimentally measurable *in vitro*, and as shown previously (Wilson and Moehlis 2014) is robust to inaccuracies. For these reasons, we believe that this methodology could be successfully tested on an *in vitro* population of neurons.

Limitations of this work include a neglect of energy considerations of processing power required to implement the control logic as detailed in the text. While there is a tremendous savings in energy injected into the brain as compared with a pulsatile stimulus, the continuous observation of the system may require a significant amount of energy, which we have not addressed here. Furthermore, we do not directly address the way in which the average transmembrane voltage of a population can be measured. We posit that the local field potential might be used as a proxy for the average voltage, as there is evidence that the two are correlated (Shimamoto A et al. 2013), but its efficacy as an observable for control problems remains to be seen. Also, in order to formulate a tractable control problem, we take the voltage probe’s effect on each neuron as the second spatial derivative of the voltage, which is an approximation of the actual effect on a more detailed compartmental model. Future studies could be performed to address the effect of this assumption in compartmental models of neural activity.

While this study provides numerical evidence that the method described in Wilson and Moehlis (2014) can be implemented with an extracellular DBS framework, further refinement may be required before it can be implemented as a treatment for Parkinson’s disease. For instance, correlated pathological bursting at a cellular level may contribute to the symptoms of Parkinson’s disease (Bevan et al. 2006). Indeed, viewing Parkinson’s disease from this perspective has led to new hypotheses about the functional mechanism of DBS. For instance, (Gale et al. 2009) posits that DBS may disrupt burst synchronization and restore neural information carrying capacity. Other works which implicate the important role that bursting plays in Parkinson’s disease include (Tai et al. 2011) which suggests that total charge delivered by DBS may be an important factor in inhibiting neural bursting and improving the symptoms of Parkinson’s disease, (Hahn et al. 2008) which reports that the amount of bursting is reduced during therapeutic DBS, and Ammari et al. (2011) which investigates the genesis of bursting in Parkinson’s disease and the effect of DBS on burst transmission. If disruption of burst synchronization is an important goal of theraputic DBS, this method could be applied to bursting models of neural activity by identifying a bursting limit cycle and subsequent PRC (Sherwood and Guckenheimer 2010).

This methodology is relatively flexible, as the cost function can be adapted as necessary to include other quantities (e.g. hardware limitations or biological considerations) that are relevant to DBS. This methodology not only has the potential to extend the implanted battery life of the DBS probe, but might also eliminate the painstaking process of manually tuning DBS parameters to suit each patient, and could represent a tremendous advance in the technology of DBS for Parkinson’s disease.

## Figures and Tables

**Fig. 11:**
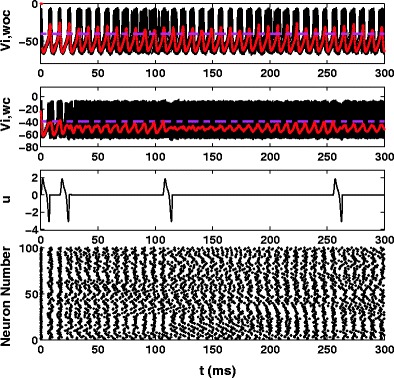
Results for a population of *N*=100 noisy neurons with electrotonic coupling and a heterogeneous distribution of distances, *d*, from the probe found using *𝜗*=0.2. The *first panel* shows results in the absence of control. The *second* and *third panels* show results for the same network with the event-based control applied. Traces give the mean voltages for the system and the horizontal line shows the control activation threshold. Substantial desynchronization can be seen from the raster plot

**Fig. 12:**
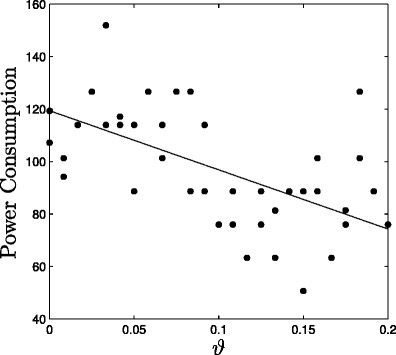
Power consumption over 1000 milliseconds is plotted for different values *𝜗*. Overall, power consumption tends to decrease as *𝜗* increases, indicating that more heterogeneity in the spatial distribution of neural distances increases the efficacy of the desynchronizing control

**Fig. 13:**
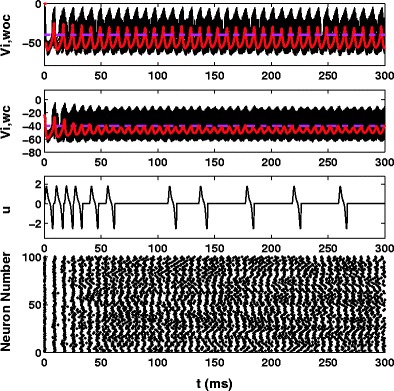
Results for a population of *N*=100 noisy neurons with synaptic coupling and a homogeneous distribution of distances from the probe. The *first panel* shows results in the absence of control. The *second* and *third panels* show results for the same network with the event-based control applied. Traces give the mean voltages for the system and the horizontal line shows the control activation threshold. Substantial desynchronization can be seen from the raster plot

**Fig. 14:**
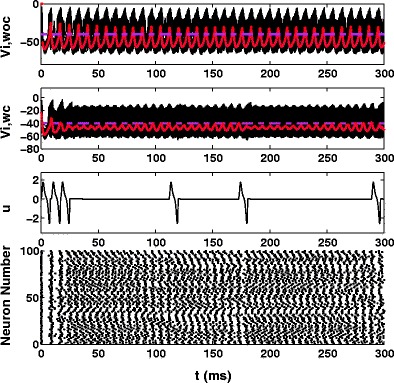
Results for a population of *N*=100 noisy neurons with synaptic coupling and a heterogeneous distribution of distances from the probe found using *𝜗*=0.2. The *first panel* shows results in the absence of control. The *second* and *third panels* show results for the same network with the event-based control applied. Traces give the mean voltages for the system and the horizontal line shows the control activation threshold. Substantial desynchronization can be seen from the raster plot

## Appendix A Network simulations with heterogeneous probe distances and electrotonic coupling

All neurons from network simulations shown in the body of the main text are assumed to be located at a distance of 1 mm from the tip of the voltage probe. Clearly, this would not be the case in a real network of neurons, and we show another network of neurons with a heterogeneous distance *d* from the voltage probe. Here, our population of neurons has a distribution of distances given by $d = 1 + \mathcal {N}(0,\vartheta ) \text {mm}$, where $\mathcal {N}(0,\vartheta )$ is a normal distribution with a mean of 0 and a standard deviation of *𝜗*. Heterogeneity in *d* is accounted for by modifying Eq. (1) as follows,

19 $$ \dot{V_{i}} = \left[ I_{m,i} + I_{ext}^{i}(t,d) + \frac{1}{N} \sum^{N}_{i =1}\sigma_{ij}(V_{j}-V_{i}) + \eta_{i}(t) \right] /C. $$

Equation (19) differs from Eq. (1) in the main text through the spatial dependence in the term $ I_{ext}^{i}(t,d)$. We calculate $ I_{ext}^{i}(t,d)$ using Eq. (5) in the main text. Here, a neuron’s distance, *d*, from the probe determines the relative strength of the external current, $\mathcal {F}(d)$, shown in Fig. 2 of the main text. We use the optimal desynchronizing stimulus associated with *γ*=8 from the text. Results for a network of *N*=100 neurons are shown in Fig. 11. Comparing with Fig. 5 from the main text, we find that for the heterogeneous case, desynchronization occurs faster, with fewer applications of the optimal stimulus

To further investigate the effect of increased heterogeneity in the spatial position of the neurons, we simulate the system for many values of *𝜗*, and after allowing the controller to initially desynchronize the neurons, we measure the power output from the controller over the next 1000 milliseconds as $\int {u_{e}^{2}} dt$. Results are given in Fig. 12. Using a linear fit to the data shows that the controller tends to use less power as the variance of the distribution in distances is increased. This phenomenon can be seen in individual simulations by comparing the applied control between simulations in Figs. 5 and 11.

## Appendix B Network simulations with excitatory synaptic coupling

Electrotonic coupling is used in the main text as a simple representation of neural coupling because it is amenable to direct calculation of its influence on the Lyapunov exponent. Here we present further simulations where neurons are synaptically coupled (Ermentrout and Terman 2010). We incorporate synaptic coupling by modifying Eq. (1) as follows:

20 $$\begin{array}{@{}rcl@{}} \dot{V_{i}} &&= \left[ I_{m,i} {}+{} I_{ext}^{i}(t,d) {}+{} \frac{(V_{G \rightarrow G}-V_{i})}{N} \sum^{N}_{j=1}g_{j}(t{}-{}t_{j})\sigma_{ij}+ \eta_{i}(t) {\vphantom{I_{m,i} + I_{ext}^{i}(t,d) + \frac{(V_{G \rightarrow G}-V_{i})}{N} \sum^{N}_{j=1}g_{j}(t-t_{j})\sigma_{ij}}}\right] /C. \end{array} $$

This equation differs from both Eq. (1) from the main text and Eq. (19) in the term $\frac {(V_{G \rightarrow G}-V_{i})}{N} \sum ^{N}_{j =1}g_{j}(t-t_{j})\sigma _{ij}$. Here, *σ*_*i**j*_=2 characterizes the synaptic coupling strength between neurons, *V*_*G*→*G*_=0 mV represents the neurotransmitter reversal potential, and *g*_*j*_(*t*−*t*_*j*_) represents a conductance-like term with *t*_*j*_ being the time that the *j* ^*t**h*^ neuron last spiked. To mimic the sudden rise and exponential decay of synaptic currents (Ermentrout and Terman 2010), we take $g_{j}(t) = \frac {t}{\tau } \exp (-t/\tau )$ where *τ*=0.3 is the synaptic time scale. We note that the synaptic coupling is all-to-all and excitatory. We use the optimal desynchronizing stimulus associated with *γ*=8 from the text. Results for a network of *N*=100 neurons, each *d*=1 *m* *m* from the voltage probe are shown in Fig. 13. The same network simulation with a distribution of distances given by $d = 1 + \mathcal {N}(0,\vartheta ) \text {mm}$, where $\mathcal {N}(0,\vartheta )$ is a normal distribution with a mean of 0 and a standard deviation of *𝜗* is shown in Fig. 14. In both simulations, the optimal control is able to sufficiently desynchronize the population, but in simulations for the network with a heterogeneous distribution of distances, the optimal control desynchronizes much more effectively.
